# Diagnostic accuracy of lung ultrasound for SARS-CoV-2: a retrospective cohort study

**DOI:** 10.1186/s13089-021-00217-7

**Published:** 2021-03-01

**Authors:** Daniel S. Brenner, Gigi Y. Liu, Rodney Omron, Olive Tang, Brian T. Garibaldi, Tiffany C. Fong

**Affiliations:** 1 Department of Emergency Medicine, Johns Hopkins School of Medicine, 1830 East Monument St Suite 6-100, Baltimore, MD 21287 USA; 2 Hospitalist Program, Division of General Internal Medicine, Department of Medicine, Johns Hopkins School of Medicine, Baltimore, MD USA; 3 Medical Scientist Training Program, Johns Hopkins School of Medicine, Baltimore, MD USA; 4 Department of Pulmonary and Critical Care Medicine, Johns Hopkins Hospital, Baltimore, MD USA

**Keywords:** POCUS, Ultrasound, COVID-19, SARS-CoV-2, Triage, Diagnosis, RT-PCR

## Abstract

**Background:**

As medical infrastructures are strained by SARS-CoV-2, rapid and accurate screening tools are essential. In portions of the world, reverse transcription polymerase chain reaction (RT-PCR) testing remains slow and in limited supply, and computed tomography is expensive, inefficient, and involves exposure to ionizing radiation. Multiple studies evaluating the efficiency of lung point-of-care ultrasound (POCUS) have been published recently, but include relatively small cohorts and often focus on characteristics associated with severe illness rather than screening efficacy. This study utilizes a retrospective cohort to evaluate the test characteristics (sensitivity, specificity, likelihood ratios, predictive values) of lung POCUS in the diagnosis of SARS-CoV-2, and to determine lung score cutoffs that maximize performance for use as a screening tool.

**Results:**

Lung POCUS examinations had sensitivity 86%, specificity 71.6%, NPV 81.7%, and PPV 77.7%. The Lung Ultrasound Score had an area under the curve of 0.84 (95% CI 0.78, 0.90). When including only complete examinations visualizing 12 lung fields, lung POCUS had sensitivity 90.9% and specificity 75.6%, with NPV 87.2% and PPV 82.0% and an area under the curve of 0.89 (95% CI 0.83, 0.96). Lung POCUS was less accurate in patients with a history of interstitial lung disease, severe emphysema, and heart failure.

**Conclusions:**

When applied in the appropriate patient population, lung POCUS is an inexpensive and reliable tool for rapid screening and diagnosis of SARS-CoV-2 in symptomatic patients with influenza-like illness. Adoption of lung POCUS screening for SARS-CoV-2 may identify patients who do not require additional testing and reduce the need for RT-PCR testing in resource-limited environments and during surge periods.

**Supplementary Information:**

The online version contains supplementary material available at 10.1186/s13089-021-00217-7.

## Introduction

A pandemic of severe acute respiratory syndrome coronavirus 2 (SARS-CoV-2), which causes the syndrome known as COVID-19 has led to over 75 million infections and over 1 million deaths as of December 2020 [1]. Early diagnosis is vital to enable early isolation, reduce ongoing transmission, and facilitate clinical decision-making. SARS-CoV-2 RT-PCR is the current diagnostic gold standard but has an estimated sensitivity of 75% [2, 3], and may take multiple days to result in some high-demand areas [4]. In the absence of sufficient RT-PCR availability, thoracic computed tomography (CT) has been used to detect the characteristic peripheral ground-glass opacities of COVID-19 pneumonia [5–8]. However, CT is not an optimal screening tool as it exposes patients to ionizing radiation, requires extensive decontamination, and is not readily available in many resource-limited situations [9–11].

Lung point-of-care-ultrasound (POCUS) has been proposed as a screening tool for COVID-19, and may offer several advantages. For non-SARS-CoV-2 interstitial syndromes, lung ultrasound has better sensitivity and specificity than chest radiograph and CT [5, 12, 13]. In confirmed cases of SARS-CoV-2 infection, lung POCUS evaluation of disease severity has correlated well with CT chest [14]. In addition to superior performance, lung POCUS is cost-effective [15, 16], portable, provides real-time data, and does not require ionizing radiation. POCUS supports infection control efforts by minimizing the number of healthcare workers exposed to patients under investigation (PUI) for COVID-19, and decontamination of ultrasound machines is relatively quick and easy compared with other imaging modalities [17]. Thus, if proven reliable, lung POCUS would allow for expedited and cost-efficient diagnosis of COVID-19 in hospitals, in the community, in resource-limited settings, and in surge situations when RT-PCR or CT chest availability is limited.

Preliminary studies have prompted the World Health Organization (WHO) to endorse ultrasound for use in the diagnosis of COVID-19, although this endorsement is noted as being based on weak preliminary evidence [18–20]. Characteristic findings of the COVID-19 syndrome on lung POCUS include a thickened or irregular pleural line, confluent B-lines, and subpleural consolidations [19, 21–23]. These findings correlate closely with those observed on CT [22], and demonstrate promise in trending clinical progression from onset to peak to resolution [21, 24]. Descriptive studies [25–31] have revealed the potential utility of lung POCUS but are limited by small sample sizes, lack of in-depth statistical analysis, and limited evaluation of patient characteristics that impact the utility of lung POCUS. To facilitate optimal application of lung POCUS for the diagnosis of COVID-19, we report the diagnostic accuracy of lung POCUS compared the criterion standard of SARS-CoV-2 RT-PCR test.

## Materials and methods

A convenience sample of COVID-19 PUI > 18 years old evaluated between March 16, 2020 and May 16, 2020 who had lung POCUS recorded as part of their routine emergency department or inpatient care were included in this retrospective cohort study conducted at two urban academic tertiary care centers. PUI designation was identified by the presence of a SARS-CoV-2 RT-PCR order by the treating physician. Criteria for SARS-CoV-2 testing during the study period included either exposure to COVID-19 or report of at least two of the following symptoms: fever, acute cough, sore throat, dyspnea, myalgias, or loss of taste or smell. Details of all patients included in the study as well as alternate diagnoses are in Additional file 1: Figure S1. This study was performed in accordance with the Declaration of Helsinki. This human study was approved by Johns Hopkins Institutional Review Board—approval: IRB00255571. Adult participant consent was not required because this was a retrospective study.

**Fig. 1:**
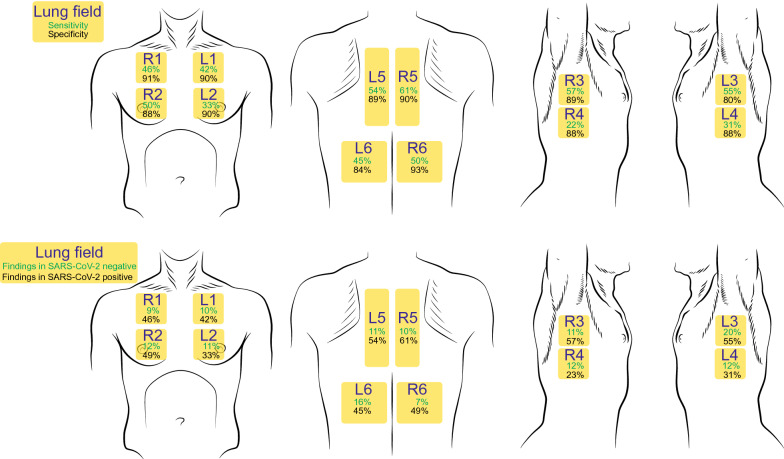
Test characteristics and distribution of findings in lung POCUS for the evaluation of SARS-CoV-2. Upper panels: Lung fields are labeled in yellow across the thorax. Green numbers represent the sensitivity and black numbers represent the specificity for SARS-CoV-2 infection of each lung field. Lower panels: Green numbers represent the percentage of patients with negative RT-PCR test for SARS-CoV-2 who had abnormal findings in each lung window. Black numbers represent the percentage of patients with positive RT-PCR test for SARS-CoV-2 who had abnormal findings in each lung window

The institutional POCUS database (Qpath Ultrasound Manager, Telexy Healthcare, Blaine WA) was queried to identify all lung POCUS examinations performed on COVID-19 PUI during the study period. All lung examinations performed during routine evaluation for COVID-19 by residents and faculty credentialed in the use of lung POCUS in the departments of Emergency Medicine and Internal Medicine were included. Study team members performed 89.1% of ultrasound studies, and all studies were evaluated for image adequacy by study team members blinded to clinical information. No dedicated training in lung POCUS or COVID-19-specific ultrasound was provided. A 12-field protocol was encouraged for POCUS users across the institution, including views of the bilateral anterior lung (L1–L2/R1–R2), lateral lung (L3–L4/R3–R4), and posterior lung (L5–L6/R5–R6) (Fig. 1). Ultrasound scans were acquired using equipment from Sonosite (Bothell WA), GE Healthcare (Waukesha WA), Philips (Bothell WA), and EchoNous (Redmond WA) (Additional file 2: Table S1).

Demographic characteristics including age, gender, race, duration of symptoms before POCUS examination, duration of symptoms before RT-PCR, RT-PCR result, body mass index (BMI), and history of comorbid conditions were recorded in a REDCap database (Vanderbilt University, Nashville TN). Two study team members credentialed in lung POCUS and blinded to all clinical information interpreted the lung POCUS studies. Reviewers did not evaluate POCUS examinations that they acquired. The blinded reviewers recorded their assessments in a separate REDCap tool based on a Lung Ultrasound Score described for use in COVID-19 pneumonia [21]. Each lung field was assessed for abnormal findings including pleural irregularity (0 points), multiple discrete B-lines (1 point), confluent B-lines (2 points), subpleural consolidations (3 points), and pleural effusion (0 points). Examples of these findings are presented in Additional file 3: Video S1, Additional file 4: Video S2, Additional file 5: Video S3, Additional file 6: Video S4, Additional file 7: Video S5, Additional file 8: Video S6. The reviewers also provided a summary assessment of whether the POCUS examination was consistent with COVID-19. A third blinded reviewer adjudicated any disagreements between reviewers. The reviewer whose ultrasound COVID-19 determination was concordant with the adjudicator was retained in the analysis.

The test characteristics and discriminative power of the Lung Ultrasound Score relative to the SARS-CoV-2 RT-PCR result were calculated. The point totals for all lung fields were summed to yield a total Lung Ultrasound Score for each reviewer. Mean Lung Ultrasound Score was calculated as the mean score between the two ultrasound reviewers. POCUS examinations were compared with the institutional gold standard RT-PCR test. A secondary analysis of complete examinations (12 acquired lung fields) was also performed. Means and proportions were compared using Student’s *T* test and Chi-squared testing. Inter-rater reliability of lung POCUS interpretation was assessed using percent agreement, percent positive agreement, and kappa statistics. The sensitivity, specificity, negative predictive value, and positive predictive value associated with overall COVID-19 status determination by ultrasound and individual lung fields were also calculated. Agreement between SARS-CoV-2 RT-PCR testing and lung POCUS was evaluated using kappa statistics. The area under the receiver operator curve was then calculated for the Lung Ultrasound Score, and a cutoff proposed based on the Youden J statistic [32]. Scoring frameworks assigning negative points for pleural effusions were assessed, and the framework with the highest discriminative power (pleural effusion − 3 points) was included for analysis.

## Results

Patients included in the cohort (*n* = 174) had mean age of 53.1 years, were 52.9% male, and 53% had positive RT-PCR testing for SARS-CoV-2 (Table 1). 77% were admitted to the hospital, 26.4% were admitted to the intensive care unit, 5.7% required high-flow nasal cannula oxygen support, and 14.4% required intubation (Table 1, Additional file 2: Table S2)**.** Other details of hospital admission and level of care are reported in Additional file 2: Table S2. Pathological lung findings were identified more frequently in all lung fields in patients who tested positive for SARS-CoV-2 RNA compared to those who tested negative (*p* < 0.001). Bilateral lung involvement was more common among those with a positive RT-PCR result (78%), compared to those with a negative RT-PCR result (26%).

**Table 1:** Baseline characteristics by SARS-CoV-2 RT-PCR results, *N* = 174

	Overall	RT-PCR result		*p* value
		Negative	Positive	
*N*	174	81	93	
Age, mean (SD)	53.1 (16.8)	51.9 (19.2)	54.2 (14.3)	0.37
Male, no. (%)	92 (53%)	40 (49%)	52 (56%)	0.39
Race, no. (%)				< 0.001
Caucasian	66 (38%)	36 (44%)	30 (32%)	
African American	33 (19%)	4 (5%)	29 (31%)	
Hispanic or Asian	75 (43%)	41 (51%)	34 (37%)	
Body mass index, kg/m^2^, mean (SD)^a^	29.1 (7.2)	28.1 (7.8)	30.0 (6.6)	0.098
Number of comorbidities, no. (%)				0.013
0	101 (58.0%)	37 (46%)	64 (69%)	
1	53 (30.5%)	30 (37%)	23 (25%)	
2	15 (8.6%)	10 (12%)	5 (5%)	
3	5 (2.9%)	4 (5%)	1 (1%)	
Interventions needed, no. (%)				
High-flow nasal cannula oxygen	10 (5.7%)	1 (1%)	9 (10%)	0.017
Intubation	25 (14.4%)	5 (6%)	20 (22%)	0.004
Comorbidities, no. (%)				
Interstitial lung disease	7 (4%)	6 (7%)	1 (1%)	0.034
Asthma	20 (12%)	8 (10%)	12 (13%)	0.53
COPD	12 (7%)	9 (11%)	3 (3%)	0.041
Heart failure	39 (22%)	27 (33%)	12 (13%)	0.001
EF ≤ 35%	11 (6%)	9 (11%)	2 (2%)	0.015
HIV/AIDS CD4 < 200	5 (3%)	4 (5%)	1 (1%)	0.13
Immunosuppression	10 (6%)	5 (6%)	5 (5%)	0.82
ESRD	5 (3%)	3 (4%)	2 (2%)	0.54
Lung Ultrasound Score, mean (SD)	6.2(5.7)	2.6 (3.2)	9.4 (5.5)	< 0.001
Positive findings by lung field, no. (%)				
L1	47 (27%)	8 (10%)	39 (42%)	< 0.001
L2	40 (23%)	9 (11%)	31 (33%)	< 0.001
L3	67 (39%)	16 (20%)	51 (55%)	< 0.001
L4	39 (22%)	10 (12%)	29 (31%)	0.003
L5	59 (34%)	9 (11%)	50 (54%)	< 0.001
L6	55 (32%)	13 (16%)	42 (45%)	< 0.001
R1	50 (29%)	7 (9%)	43 (46%)	< 0.001
R2	56 (32%)	10 (12%)	46 (49%)	< 0.001
R3	62 (36%)	9 (11%)	53 (57%)	< 0.001
R4	31 (18%)	10 (12%)	21 (23%)	0.078
R5	65 (37%)	8 (10%)	57 (61%)	< 0.001
R6	52 (30%)	6 (7%)	46 (49%)	< 0.001
Days between symptom onset and test				
RT-PCR, median (IQR)	3 (2, 7)	3 (2, 5)	5, (3, 7)	< 0.001
POCUS, median (IQR)	6 (3, 10)	3 (2, 6)	8 (6, 14)	< 0.001
Extent of lung findings, no. (%)				
No findings	53 (31%)	42 (52%)	11 (12%)	< 0.001
Single field involvement	16 (9%)	102 (15%)	4 (4%)	
Multiple unilateral field involvement	11 (6%)	6 (7%)	5 (5%)	
Bilateral field involvement	94 (54%)	21 (26%)	73 (78%)	

*EF* ejection fraction, *HIV/AIDS* human immunodeficiency virus/acquired immunodeficiency syndrome, *ESRD* end-stage renal disease, *IQR* interquartile range

^a^6.9% missing

Test characteristics for lung POCUS in the diagnosis of COVID-19 are detailed in Table 2. Compared to the standard of RT-PCR testing, lung POCUS had a sensitivity 86.0% and specificity 71.6%, with negative predictive value (NPV) 81.7% and positive predictive value (PPV) 77.7%. Test characteristics for the involvement of more than one lung field, posterior lung field involvement, and bilateral lung field involvement are also reported in Table 2. Examinations with multiple discrete B-lines had a sensitivity 86.0%, specificity 54.3%, NPV 77.2%, and PPV 68.4% while examinations with confluent B-lines had a sensitivity 43.0%, specificity 98.8%, NPV 60.2%, PPV 97.6%. Lung POCUS was more accurate in patients with more significant oxygen requirement or requiring higher level of care (Table 3).

**Table 2:** Sensitivity, specificity, negative predictive value (NPV), positive predictive value (PPV), and kappa statistics associated with lung ultrasound findings compared to SARS-CoV-2 RT-PCR testing

	Sensitivity	Specificity	NPV	PPV	Kappa
All ultrasounds, *N* = 174					
Ultrasound SARS-CoV-2 diagnosis	86.0	71.6	81.7	77.7	0.58
Multiple field involvement	83.9	66.7	78.3	74.3	0.51
Posterior field involvement	71.0	70.4	67.9	73.3	0.41
Bilateral lung involvement	78.5	74.1	75.0	77.7	0.53
Bilateral posterior field involvement	57.0	91.4	64.9	88.3	0.47
Any discrete B-lines	86.0	54.3	77.2	68.4	0.41
Any confluent B-lines	43.0	98.8	60.2	97.6	0.40
Any pleural thickening or irregularity	86.0	51.9	76.4	67.2	0.39
No pathologic lung findings	11.8	48.1	32.2	20.8	-0.39
Ultrasound SARS-CoV-2 diagnosis					
No ILD, *N* = 167	87.0	77.3	82.9	82.5	0.65
No ILD, no HF EF ≤ 35%, *N* = 158	85.4	82.4	83.6	86.8	0.70
No ILD, no HF EF ≤ 35%, no immunosuppression, *N* = 144	89.3	83.3	84.7	88.2	0.73
Complete ultrasounds, *N* = 174	90.9	75.6	87.2	82.0	0.67

*ILD* interstitial lung disease, *EF* ejection fraction, *HIV/AIDS* human immunodeficiency virus/acquired immunodeficiency syndrome, *ESRD* end-stage renal disease

**Table 3:** Sensitivity, specificity, negative predictive value (NPV), positive predictive value (PPV), and kappa statistics by disposition and oxygen requirement

	Sensitivity	Specificity	NPV	PPV	Kappa
Disposition					
Discharged from ED, *N* = 41	76.9	89.3	89.3	76.9	0.66
Floor/IMC, *N* = 86	82.9	64.4	80.6	68.0	0.47
ICU, *N* = 46	92.1	50.0	57.1	89.7	0.44
Oxygen requirement					
None or nasal canula, *N* = 139	84.4	73.3	84.6	73.0	0.57
HFNC, *N* = 10	88.9	100.0	50.0	100.0	0.62
Intubation, *N* = 25	90.0	40.0	50.0	85.7	0.32

**Fig. 2:**
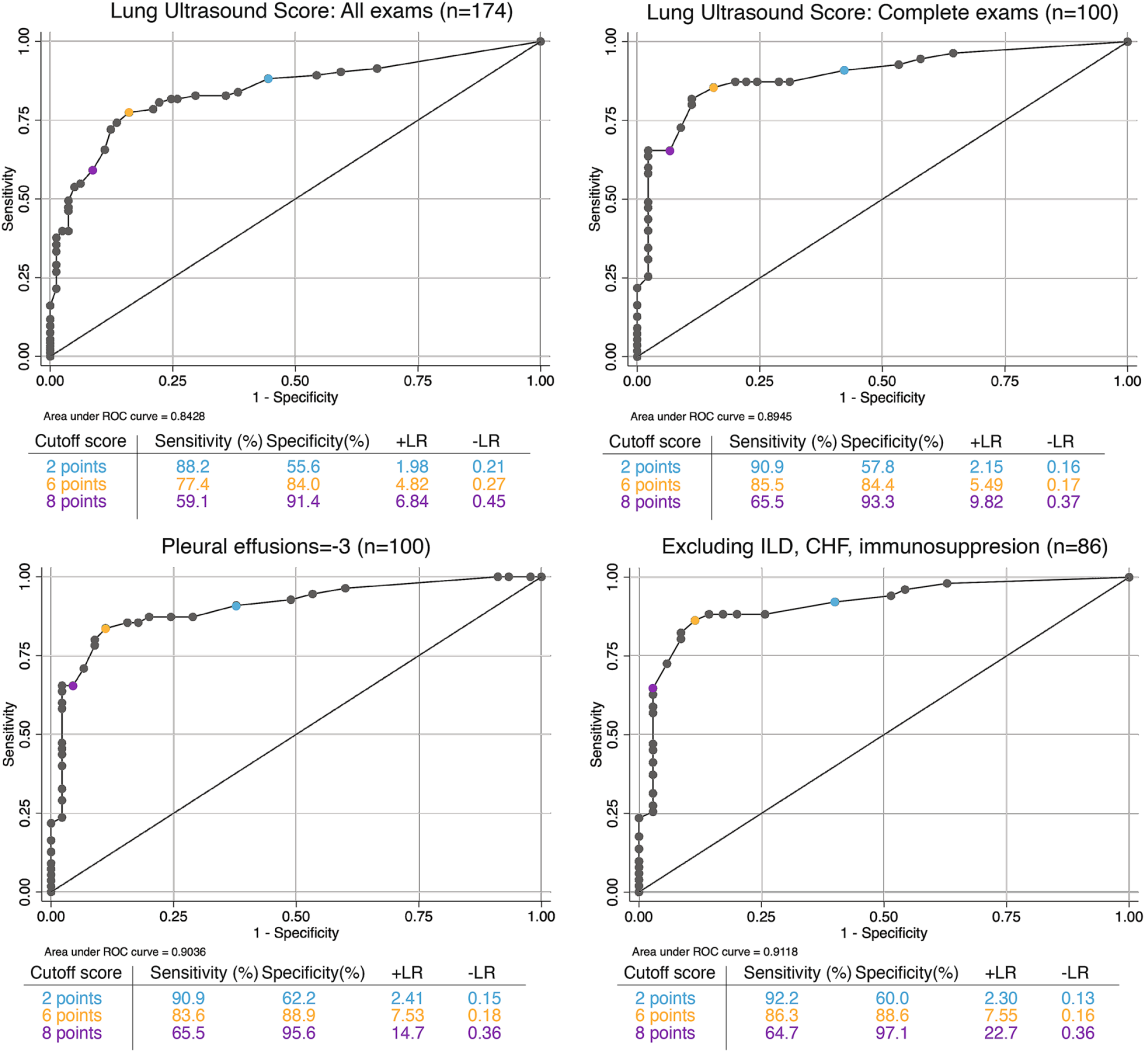
Receiver operator curves for Lung Ultrasound Score for all examinations (top left), examinations with all 12 views (top right), examinations with 12 views including pleural effusions as a negative prognostic factor for SARS-CoV-2 diagnosis (bottom left), and excluding confounding pre-existing comorbidities (bottom right)

**Fig. 3:**
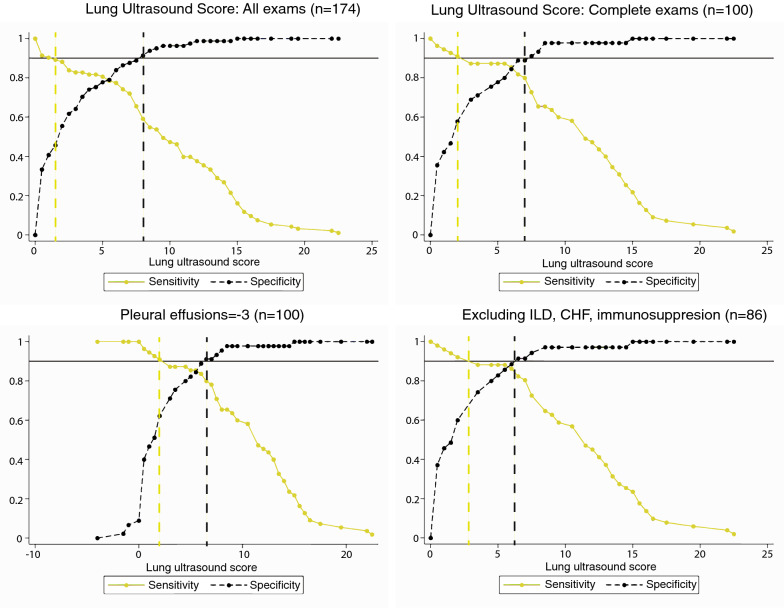
Grey zone analysis for Lung Ultrasound Score for all examinations (top left), examinations with all 12 views (top right), examinations with 12 views including pleural effusions as a negative prognostic factor for SARS-CoV-2 diagnosis (bottom left), and excluding confounding pre-existing comorbidities (bottom right)

Patients with positive SARS-CoV-2 RT-PCR testing had higher Lung Ultrasound Scores than those with negative tests (9.4 ± 5.5 versus 2.6 ± 3.2). There was high discrimination (AUC 0.84, 95% CI 0.78, 0.90) of the Lung Ultrasound Score for COVID-19 syndrome (Fig. 2). The test characteristics associated with various cutoff points are reported in Fig. 2. A Lung Ultrasound Score of 2 points maximized sensitivity (sensitivity 88%, specificity 55%, positive likelihood ratio (+ LR) 1.98, negative likelihood ratio (− LR) 0.21, PPV 80.4**%,** NPV 69.5%). A Lung Ultrasound Score of 8 points maximized specificity (sensitivity 59%, specificity 91%, + LR 6.84, − LR 0.45, PPV 66.1%, NPV 88.7%). A Lung Ultrasound Score of 6 points optimized a balance of sensitivity and specificity (sensitivity 77%, specificity 84%, + LR 4.82, − LR 0.27, PPV 76.4%, NPV 84.7%) are also reported in Fig. 2. A grey zone analysis in Fig. 3 demonstrates that Lung Ultrasound Scores under 4 have sensitivity over 90% of SARS-CoV-2 infection, and that scores over 6 have over 90% specificity for the detection of SARS-CoV-2 infection. A modified version of the Lung Ultrasound Score that scored the presence of pleural effusion as − 3 points had improved discrimination compared to the standard Lung Ultrasound Score (Fig. 2; AUC 0.90, 95% CI 0.84, 0.96), and improved test characteristics (Fig. 2). Using pleural effusions as a negative prognostic factor improved the performance of the Lung Ultrasound Score in the grey zone analysis, narrowing the area of uncertain diagnosis (Fig. 3).

Patient-related factors associated with decreased lung POCUS accuracy were also investigated. Patients with false-negative lung POCUS scans (*n* = 13) had higher average BMI compared to patients with true-positive scans (36.5 kg/m^2^ ± 9.4 versus 29.3 kg/m^2^ ± 8.1; *p* < 0.001) (Table 4). Patients with false-positive lung POCUS scans (*n* = 23) were more likely to have pre-existing ILD than patients with true negative scans (26% versus 0%, *p* < 0.001; Table 4). Patients with false-positive lung POCUS scans were also more likely to have systolic heart failure (57% versus 24% *p* < 0.001) and heart failure with ejection fraction (EF) under 35% (30% vs 3% *p* < 0.001; Table 4). There was no difference in the prevalence of asthma, COPD, HIV with CD4 < 200, immunosuppression, or end-stage renal disease in patients with discordance between lung POCUS and RT-PCR result (Table 4).

**Table 4:** Factors associated with concordance or discordance between SARS-CoV-2 RT-PCR and POCUS diagnosis of SARS-CoV-2

SARS-CoV-2 RT-PCR	Negative	Positive	Negative	Positive	*p* value
POCUS SARS-CoV-2	Negative	Negative	Positive	Positive	
*N*	58	13	23	80	
Body mass index, kg/m^2^, mean (SD)^a^	29.3 (8.1)	36.5 (9.4)	25.4 (6.3)	29.1 (5.6)	< 0.001
Number of lung fields visualized, median (IQR)	12 (8, 12)	11 (10, 12)	11 (9, 12)	12 (10, 12)	0.38
Lung Ultrasound Score, mean (SD)	1.1 (1.6)	0.6 (0.9)	6.3 (3.2)	10.8 (4.5)	< 0.001
Days between symptom onset and test					
POCUS, median (IQR)	3 (2, 5)	6 (4, 8)	3 (2, 6)	9.0 (6.0, 14.0)	< 0.001
RT-PCR, median (IQR)	0 (0, 0)	3.0 (0, 5)	0 (0, 1)	2 (0, 6)	< 0.001
Number of comorbidities, no (%)					
0	32 (55%)	7 (54%)	5 (22%)	57 (71%)	0.041
1	19 (33%)	4 (31%)	11 (48%)	19 (24%)	
2	7 (12%)	1 (8%)	3 (13%)	4 (5%)	
3	0 (0%)	1 (8%)	4 (17%)	0 (0%)	
Comorbidities, no (%)					
Interstitial lung disease	0 (0%)	1 (8%)	6 (26%)	0 (0%)	< 0.001
Asthma	6 (10%)	1 (8%)	2 (9%)	11 (14%)	0.84
COPD (without severe emphysematous changes)	6 (10%)	1 (8%)	3 (13%)	2 (2%)	0.18
Heart failure	14 (24%)	4 (31%)	13 (57%)	8 (10%)	< 0.001
EF ≤ 35%	2 (3%)	1 (8%)	27 (30%)	1 (1%)	< 0.001
HIV/AIDS CD4 < 200	2 (3%)	0 (0%)	2 (9%)	1 (1%)	0.26
Immunosuppression	4 (7%)	2 (15%)	1 (4%)	3 (4%)	0.39
ESRD	1 (2%)	0 (0%)	2 (9%)	2 (2%)	0.32

*EF* ejection fraction, *HIV/AIDS* human immunodeficiency virus/acquired immunodeficiency syndrome, *ESRD* end-stage renal disease, *IQR* interquartile range

^a^7% missing

Exclusion of patients with these comorbid conditions associated improved the diagnostic performance of lung POCUS for COVID-19 (Table 2). In patients without these confounding conditions, the sensitivity was 89.3%, specificity was 83.3%, NPV 84.7%, and PPV 88.2%. These exclusions also improved the discriminative ability of the Lung Ultrasound Score (Fig. 2; AUC 0.91, 95% CI 0.85, 0.98) and narrowed the grey zone area of uncertain diagnosis (Fig. 3). The test characteristics associated with cutoff points maximizing sensitivity (2 points: PPV 84.0%), specificity (8 points: NPV 97.1%), or statistically optimizing a balance of sensitivity and specificity (6 points: PPV 81.6%, NPV 91.7%) are also reported in Fig. 2.

A secondary analysis including only complete lung POCUS examinations with all 12 lung fields was performed. Participants with complete POCUS lung evaluation (*n* = 100) had a mean age of 51.3 years, were 54% male, and 55% had positive RT-PCR testing for SARS-CoV-2 (Additional file 2: Table S3). Complete examinations had improved test characteristics and better discriminative ability (Additional file 9: Figure S2) compared to examinations that did not include all lung fields (AUC 0.89, 95% CI 0.83, 0.96. Sensitivity 90.9%, specificity 75.6%, NPV 87.2%, PPV 82.0%) (Fig. 2).

## Discussion

These data demonstrate that lung POCUS provides rapid information regarding COVID-19 status that is consistent with results of the current gold standard RT-PCR test. In our patient cohort, there was also anecdotal evidence that lung POCUS could outperform the RT-PCR test. At least three patients in this study with initially negative RT-PCR testing but lung POCUS examinations suggestive of COVID-19 were subsequently diagnosed with SARS-CoV-2 infection through more invasive testing.

While descriptive studies of lung POCUS findings in COVID-19 are numerous [19, 22, 25], this is most thorough investigation of a quantitative lung POCUS score to diagnose COVID-19 with data allowing maximization of sensitivity, specificity, and discriminative ability. Other studies do provide information on the test characteristics of lung POCUS in the diagnosis of COVID-19, but are limited by much smaller cohorts and unable to demonstrate the performance in populations with various comorbidities [26, 27, 29, 31]. The present study provides a more comprehensive assessment of the diagnostic power of lung POCUS in a large heterogenous population, and provides vital information for properly applying lung POCUS in the diagnosis of COVID-19.

These data suggest that a Lung Ultrasound Score cutoff of 2 points maximizes sensitivity for use in screening of symptomatic patients. Using this approach, any POCUS examination with three or more discrete B-lines in two distinct lung fields, or any examination with confluent B-lines or subpleural consolidation in any single lung field is concerning for COVID-19, requires isolation, and may benefit from additional testing. Any lung POCUS examination with a Lung Ultrasound Score of 0 points or 1 point is very unlikely to be associated with COVID-19, and can be triaged out of PUI workflows and investigated for other etiologies of their symptoms. At any point during the examination, if the cumulative Lung Ultrasound Score is equal to or greater than 2 points, the clinician can stop and order confirmatory testing, and move on to the next patient. This approach deliberately maximizes the sensitivity and negative predictive value at the expense of the specificity and positive predictive value, and will need to be externally validated. Additionally, while this approach may be advantageous for efficiency, the secondary analysis strongly suggests that complete examinations with 12 lung fields have improved diagnostic power compared to abbreviated examinations. Other cutoffs maximizing specificity (8 points) or balancing between sensitivity and specificity (6 points) can be considered in the appropriate clinical contexts.

The accuracy of lung POCUS may be impacted by patient factors. Elevated BMI reduced the sensitivity of lung POCUS for COVID-19, consistent with prior studies that have reported similar effects of obesity [33]. Pre-existing interstitial lung disease reduced the specificity of lung POCUS for COVID-19. This confounder may be due to the pre-existing pathology of interstitial inflammation, scarring, and thickening leading to a similar ultrasonographic appearance. It is also challenging to differentiate between pulmonary edema due to heart failure [34] or end-stage renal disease and the interstitial inflammation caused by COVID-19. In patients with B-lines on lung POCUS, a concurrent cardiac POCUS may help differentiate between COVID-19 and cardiac etiologies [35]. Other tools such as M-mode evaluation for pleural irregularities [35] and the presence of "spared areas" [36] have been used to differentiate interstitial syndrome from pulmonary edema in the past, but have not been validated for use with COVID-19. Avoiding the use of lung POCUS in patients with these confounding comorbidities (morbid obesity, interstitial lung disease, heart failure) improves the diagnostic performance for COVID-19.

### Study limitations

The retrospective design predisposes to recruitment bias. This study is somewhat insulated from this limitation since the RT-PCR testing often had not resulted when the POCUS images were acquired and when the patients were identified for inclusion in the review. The inclusion of patients with variable duration of symptoms and illness severity as well as in different practice environments raises concerns for spectrum bias, but also demonstrates the accuracy that can be expected with real-world application of this diagnostic test.

The majority (77%) of the patients evaluated in this study were admitted to the hospital, which may limit the applicability of these findings in patients who do not require hospitalization. Several factors contribute to this bias, including triage of less severe, outpatient-appropriate patients to a treatment tent without ultrasound equipment, absence of clear guidelines for safe discharge early in the pandemic, and the nature of the tertiary hospital study site as a transfer center for care of the majority of admitted COVID-19 patients in the health system. Despite the preponderance of hospitalized patients, the variable lengths of stay (interquartile range 1–11 days) hint at a wide spectrum of illness severity and resource requirement and suggest that the lung POCUS findings may be broadly generalizable.

Another limitation is the dependence on POCUS operator skill and experience. Less-experienced point-of-care ultrasonographers tended to more aggressively label mild abnormalities in a single lung field as evidence COVID-19 even though the data suggest that most patients with positive SARS-CoV-2 RT-PCR tests have bilateral pathological findings. Undergained or overgained images could also lead to false-negative or false-positive POCUS interpretations, although all images for this study were assessed for quality prior to inclusion. Mimics such as Z-lines (short, comet-tail artifacts arising from the pleural line that do not reach the distal end of the screen or erase A-lines) [37] and E-lines (long comet-tail artifacts that do erase A-lines but arise from the subcutaneous tissue rather than the pleura) [37] can easily be confused with B-lines and lead to false-positive diagnoses. The development of training resources will be crucial for widespread implementation of lung POCUS as a screening tool for COVID-19.

As this study only included PUI for COVID-19, it is unknown whether lung POCUS can be used as a screening tool for asymptomatic SARS-CoV-2 carriers. It is also unclear whether lung POCUS can be used for the diagnosis of COVID-19 in patients with primarily gastrointestinal [38] or neurological [39] symptoms. Additional studies will be necessary before lung POCUS can be applied for the screening and diagnosis of COVID-19 in these situations.

The results of this study are also applicable only during the current clinical environment, with high prevalence of SARS-CoV-2. In future periods with lower prevalence, lung POCUS may not perform well enough to use as a screening tool.

Finally, the use of RT-PCR as the reference standard is a significant limitation. Although RT-PCR is currently the gold standard for diagnosis, its sensitivity is known to be relatively limited [2]. Future, prospective studies will be needed to test whether lung POCUS provides improved sensitivity over RT-PCR testing in certain circumstances.

## Conclusions

Lung POCUS is a rapid, inexpensive tool that provides results that are concordant with RT-PCR testing in patients under investigation for COVID-19. The low cost, rapid assessment, lack of ionizing radiation, and applicability to a variety of practice environments make it an appealing option for use when other diagnostic tests such RT-PCR or CT chest are unavailable.

## Supplementary Information

**Additional file 1: Figure S1.** Flowsheet of patients that were included, results of testing, and associated confounding factors.

**Additional file 2:** **Table S1**. Ultrasound machines, probes, and parameters. **Table S2**. Disposition of included patients. **Table S3**. Characteristics of ultrasound cohort with complete examinations (*N* = 100). **Table S4.** Characteristics of ultrasound cohort with complete examinations (*N* = 100) by ultrasound and RT-PCR SARS-CoV-2 diagnosis.

**Additional file 3: Video S1.** Normal lung POCUS findings.

**Additional file 4: Video S2.** Lung POCUS findings from a patient diagnosed with COVID-19, including discrete B-lines, confluent B-lines, irregular pleural line, and small subpleural consolidations

**Additional file 5:** **Video S3.** Lung POCUS findings from a patient diagnosed with COVID-19, including subpleural consolidation.

**Additional file 6: Video S4.** Lung POCUS findings from a patient with CHF consistent with cardiogenic pulmonary edema. These findings lead to a false-positive lung POCUS interpretation.

**Additional file 7: Video S5. ** Lung POCUS findings demonstrating a large pleural effusion from a patient with heart failure.

**Additional file 8:** **Video S6.** Lung POCUS findings from a patient with interstitial lung disease. These findings lead to a false-positive lung POCUS interpretation.

**Additional file 9: Figure S2.** Test characteristics and distribution of findings in complete 12-field lung POCUS examinations for the evaluation of SARS-CoV-2. Upper panels: Lung fields are labeled in yellow across the thorax. Green numbers represent the sensitivity and black numbers represent the specificity for SARS-CoV-2 infection of each lung field. Lower panels: Green numbers represent the percentage of patients with negative RT-PCR test for SARS-CoV-2 who had abnormal findings in each lung window. Black numbers represent the percentage of patients with positive RT-PCR test for SARS-CoV-2 who had abnormal findings in each lung window.

## Data Availability

The dataset used during the current study are available from the corresponding author on reasonable request, subject to approval from the institutional review board.

## Abbreviations

POCUS: Point of care ultrasound
SARS-CoV-2: Severe Acute Respiratory Syndrome Coronavirus 2
RT-PCR: Reverse transcription polymerase chain reaction
CT: Computed tomography
PUI: Patient under investigation
+LR: Positive likelihood ratio
-LR: Negative likelihood ratio
PPV: Positive predictive value
NPV: Negative predictive value
AUC: Area under the curve
HIV: Human immunodeficiency virus
BMI: Body mass index
COPD: Chronic obstructive pulmonary disease
ESRD: End stage renal disease
