# Supplementary material for: Diagnostic accuracy of lung ultrasound for SARS-CoV-2: a retrospective cohort study
Source: Ultrasound J. 2021 Mar 1;13:12. doi: 10.1186/s13089-021-00217-7 (PMC7916995; doi:10.1186/s13089-021-00217-7)
Supplement: Supplementary file 2 — Additional file 2: Table S1. Ultrasound machines, probes, and parameters. Table S2. Disposition of included patients. Table S3. Characteristics of ultrasound cohort with complete examinations (N = 100). Table S4. Characteristics of ultrasound cohort with complete examinations (N = 100) by ultrasound and RT-PCR SARS-CoV-2 diagnosis. [file 13089_2021_217_MOESM2_ESM.docx]

Additional file:

2. **Table S1**. Ultrasound machines, probes, and parameters

3. **Table S2**. Disposition of included patients.

4. **Table S3**. Characteristics of ultrasound cohort with complete examinations (N=100)

5. **Table S4.** Characteristics of ultrasound cohort with complete examinations (N = 100) by ultrasound and RT-PCR SARS-CoV-2 diagnosis.

**Table S1**. Ultrasound probe and parameters

| NP Swab SARS-CoV-2 Result | Negative | Positive | Negative | Positive |  |
| --- | --- | --- | --- | --- | --- |
| Ultrasound SARS-CoV-2 Result | Negative | Negative | Positive | Positive | p-value |
| N | 58 | 13 | 23 | 80 |  |
| Number of fields obtained |  |  |  |  |  |
| <6 | 0 (0%) | 0 (0%) | 2 (9%) | 1 (1%) | 0.045 |
| 6-8 | 18 (31%) | 3 (23%) | 5 (22%) | 14 (18%) |  |
| 9-11 | 6 (10%) | 5 (38%) | 5 (22%) | 15 (19%) |  |
| 12 | 34 (59%) | 5 (38%) | 11 (48%) | 50 (62%) |  |
| Lung probe setting | 23 (40%) | 8 (62%) | 11 (48%) | 50 (62%) | 0.053 |
| Ultrasound probe |  |  |  |  |  |
| GE Venue | 10 (17%) | 5 (38%) | 3 (13%) | 11 (14%) | <0.001 |
| Kosmos | 0 (0%) | 0 (0%) | 1 (4%) | 2 (2%) |  |
| Philips Sparq | 2 (3%) | 1 (8%) | 1 (4%) | 0 (0%) |  |
| Phillips Lumify | 2 (3%) | 2 (15%) | 4 (17%) | 35 (44%) |  |
| Phillips Sparq | 0 (0%) | 0 (0%) | 1 (4%) | 0 (0%) |  |
| Sonosite Edge | 6 (10%) | 0 (0%) | 1 (4%) | 2 (2%) |  |
| Sonosite S2 | 9 (16%) | 3 (23%) | 5 (22%) | 9 (11%) |  |
| Sonosite Xporte | 29 (50%) | 2 (15%) | 7 (30%) | 21 (26%) |  |

**Table S2**. Disposition of included patients.

| Disposition, no. (%) | Overall | RT-PCR negative | RT-PCR positive | p-value |
| --- | --- | --- | --- | --- |
| Discharge from ED | 41 (23.6%) | 28 (35%) | 13 (14.0%) | 0.001 |
| Admitted to hospital | 134 (77.0%) | 53 (65.0%) | 81 (87.0%) | <0.001 |
| Medicine floor | 84 (48.3%) | 43 (53.0%) | 41 (44.0%) | 0.24 |
| Stepdown unit | 2 (1.1%) | 2 (2.0%) | 0 (0.0%) | 0.13 |
| ICU | 46 (26.4%) | 8 (10.0%) | 38 (41.0%) | <0.001 |
| Death during hospitalization | 6 (3.4%) | 3 (4.0%) | 3 (3.0%) | 0.84 |
| Duration of admission, median (IQR) | 5 (1, 11) | 2 (0, 6) | 8 (3, 13) | <0.001 |

**Table S3**. Characteristics of full ultrasound cohort, N= 100

|  |  | RT-PCR result | | |
| --- | --- | --- | --- | --- |
|  | Overall | Negative | Positive | p-value |
| N | 100 | 45 | 55 |  |
| Age, mean (SD) | 51.3 (17.5) | 47.9 (20.3) | 54.1 (14.3) | 0.073 |
| Male, no. (%) | 54 (54%) | 23 (51%) | 31 (56%) | 0.6 |
| Race, no. (%) |  |  |  | <0.001 |
| Caucasian | 27 (27%) | 14 (31%) | 13 (24%) |  |
| African American | 25 (25%) | 2 (4%) | 23 (42%) |  |
| Hispanic or Asian | 48 (48%) | 29 (64%) | 19 (35%) |  |
| Body mass index, kg/m^2^, mean (SD)* | 29.5 (6.0) | 28.0 (5.9) | 30.7 (5.8) | 0.026 |
| Number of comorbidities, no. (%) | |  |  | 0.31 |
| 0 | 63 (63.0%) | 24 (53%) | 39 (71%) |  |
| 1 | 29 (29.0%) | 16 (36%) | 13 (24%) |  |
| 2 | 6 (6.0%) | 4 (9%) | 2 (4%) |  |
| 3 | 2 (2.0%) | 1 (2%) | 1 (2%) |  |
| Comorbidities, no. (%) |  |  |  |  |
| Interstitial lung disease | 3 (3%) | 2 (4%) | 1 (2%) | 0.44 |
| Asthma | 10 (10%) | 5 (11%) | 5 (9%) | 0.74 |
| COPD | 5 (5%) | 2 (4%) | 3 (5%) | 0.82 |
| Heart Failure | 18 (18%) | 11 (24%) | 7 (13%) | 0.13 |
| EF ≤ 35% | 3 (3%) | 3 (7%) | 0 (0%) | 0.05 |
| HIV/AIDS CD4<200 | 4 (4%) | 3 (7%) | 1 (2%) | 0.22 |
| Immunosuppression | 5 (5%) | 3 (7%) | 2 (4%) | 0.49 |
| ESRD | 2 (2%) | 1 (2%) | 1 (2%) | 0.89 |
| Lung Ultrasound Score, mean (SD) | 6.9 (6.0) | 2.4 (3.1) | 10.6 (5.3) | <0.001 |
| Findings by lung field, no. (%) | |  |  |  |
| L1 | 23 (23%) | 1 (2%) | 22 (40%) | <0.001 |
| L2 | 25 (25%) | 4 (9%) | 21 (38%) | <0.001 |
| L3 | 40 (40%) | 9 (20%) | 31 (56%) | <0.001 |
| L4 | 27 (27%) | 7 (16%) | 20 (36%) | 0.020 |
| L5 | 45 (45%) | 6 (13%) | 39 (71%) | <0.001 |
| L6 | 46 (46%) | 10 (22%) | 36 (65%) | <0.001 |
| R1 | 25 (25%) | 2 (4%) | 23 (42%) | <0.001 |
| R2 | 29 (29%) | 1 (2%) | 28 (51%) | <0.001 |
| R3 | 37 (37%) | 4 (9%) | 33 (60%) | <0.001 |
| R4 | 15 (15%) | 5 (11%) | 10 (18%) | 0.32 |
| R5 | 47 (47%) | 4 (9%) | 43 (78%) | <0.001 |
| R6 | 46 (46%) | 5 (11%) | 41 (75%) | <0.001 |
| Days between symptom onset and test |  |  |  |  |
| RT-PCR, median (IQR) | 4.0 (2.0, 7.0) | 3.0 (2.0, 5.0) | 6.0 (3.0, 8.0) | <0.001 |
| POCUS, median (IQR) | 6.5 (3.0, 13.5) | 3.0 (2.0, 6.0) | 9.0 (6.0, 16.0) | <0.001 |
| Extent of lung findings, no. (%) | |  |  |  |
| No findings | 28 (28%) | 24 (53%) | 4 (7%) | <0.001 |
| Single field involvement | 10 (10%) | 8 (18%) | 2 (4%) |  |
| Multiple unilateral field involvement | 5 (5%) | 3 (7%) | 2 (4%) |  |
| Bilateral field involvement | 57 (57%) | 10 (22%) | 47 (85%) |  |

* 7% missing

Abbreviations: EF, ejection fraction; HIV/AIDS, human immunodeficiency virus/acquired immunodeficiency syndrome; ESRD, end-stage renal disease; IQR, interquartile range

**Table S4.** Test characteristics of ultrasound cohort with complete exams (N = 100) by ultrasound and RT-PCR SARS-CoV-2 diagnosis.

| SARS-CoV-2 RT-PCR | Negative | Positive | Negative | Positive | p-value |
| --- | --- | --- | --- | --- | --- |
| POCUS SARS-CoV-2 | Negative | Negative | Positive | Positive |  |
| N | 34 | 5 | 11 | 50 |  |
| Body mass index, kg/m^2^, mean (SD)* | 28.1 (6.4) | 46.0 (3.7) | 27.4 (4.0) | 29.4 (3.8) | <0.001 |
| Lung Ultrasound Score, mean (SD) | 1.3 (1.9) | 0.6 (0.7) | 5.9 (3.7) | 11.6 (4.5) | <0.001 |
| Lung ultrasound setting, no (%) | 15 (44%) | 5 (100%) | 7 (64%) | 38 (76%) | 0.008 |
| Days between symptom onset and test |  |  |  |  |  |
| Ultrasound, median (IQR) | 3.0 (2.0, 6.0) | 7.0 (1.0, 10.0) | 3.0 (2.0, 6.0) | 9.0 (7.0, 16.0) | <0.001 |
| RT-PCR, median (IQR) | 0.0 (0.0, 0.0) | 4.0 (0.0, 5.0) | 0.0 (0.0, 1.0) | 2.0 (0.0, 6.0) | <0.001 |
| Number of comorbidities, no (%) |  |  |  |  |  |
| 0 | 20 (59%) | 3 (60%) | 4 (36%) | 36 (72%) | 0.041 |
| 1 | 11 (32%) | 1 (20%) | 5 (45%) | 12 (24%) |  |
| 2 | 3 (9%) | 0 (0%) | 1 (9%) | 2 (4%) |  |
| 3 | 0 (0%) | 1 (20%) | 1 (9%) | 0 (0%) |  |
| Comorbidities, no (%) |  |  |  |  |  |
| Interstitial lung disease | 0 (0%) | 1 (20%) | 2 (18%) | 0 (0%) | <0.001 |
| Asthma | 4 (12%) | 0 (0%) | 1 (9%) | 5 (10%) | 0.88 |
| COPD (without severe emphysematous changes) | 2 (6%) | 1 (20%) | 0 (0%) | 2 (4%) | 0.38 |
| Heart Failure | 6 (18%) | 2 (40%) | 5 (45%) | 5 (10%) | 0.024 |
| EF ≤ 35% | 1 (3%) | 0 (0%) | 2 (18%) | 0 (0%) | 0.015 |
| HIV/AIDS CD4<200 | 2 (6%) | 0 (0%) | 1 (9%) | 1 (2%) | 0.62 |
| Immunosuppression | 3 (9%) | 0 (0%) | 0 (0%) | 2 (4%) | 0.57 |
| ESRD | 0 (0%) | 0 (0%) | 1 (9%) | 1 (2%) | 0.31 |

*7% missing
Abbreviations: EF, ejection fraction; HIV/AIDS, human immunodeficiency virus/acquired immunodeficiency syndrome; ESRD, end-stage renal disease; IQR, interquartile range
